# Collagen-Based Delivery Systems for the Prevention of Alveolitis: A Narrative Review and Rationale for Biopharmaceutical Development Requirements

**DOI:** 10.3390/jfb17020092

**Published:** 2026-02-13

**Authors:** Hadi Darawsheh, Marina M. Shumkova, Liliya M. Buraya, Maria V. Pomytkina, Ghazaleh Pouya, Farida Bakieva, Mikhail Grin, Elena O. Bakhrushina, Natalia Kireeva, Sergey Dydykin, Yuriy Vasil’ev

**Affiliations:** 1 N.V. Sklifosovskiy Institute of Clinical Medicine, First Moscow State Medical University (Sechenov University), 119435 Moscow, Russia; hadi.darawsheh@gmail.com (H.D.); kireeva_n_v_1@staff.sechenov.ru (N.K.); dydykin_s_s@staff.sechenov.ru (S.D.); 2 A.P. Nelyubin Institute of Pharmacy, First Moscow State Medical University (Sechenov University), 119435 Moscow, Russia; shumkova_m_m@staff.sechenov.ru (M.M.S.); motigadzh@mail.ru (L.M.B.); pomytkina_m_v@staff.sechenov.ru (M.V.P.); puya_g@student.sechenov.ru (G.P.); bakhrushina_e_o@staff.sechenov.ru (E.O.B.); 3 Moscow Regional Research Clinical Institute named after M. F. Vladimirsky, 129090 Moscow, Russia; fora.1506@mail.ru; 4 Lomonosov Institute of Fine Chemical Technologies, MIREA—Russian Technological University, 125993 Moscow, Russia; grin@mirea.ru

**Keywords:** collagen, alveolitis, prevention, dentistry, bone tissue, inflammatory processes

## Abstract

Alveolitis remains a common postoperative complication following tooth extraction, characterized by inflammation and delayed socket healing. Collagen-based materials have shown promise in promoting tissue regeneration and reducing inflammation. This review evaluates the efficacy of collagen in the prevention of alveolitis, with a focus on the development and application of topical delivery systems such as gels and collagen sponges. Special attention is given to the local application of these systems within the extraction socket and their performance under oral conditions. The study analyzes current evidence on the pathogenesis of alveolitis, the biological properties of collagen relevant to wound healing, and pharmaceutical strategies for enhancing its clinical effectiveness. The findings support the feasibility of using biodegradable, site-specific collagen-based formulations for alveolitis prevention. Such systems may provide a prolonged therapeutic effect, stabilize blood clots, reduce microbial contamination, and support angiogenesis and osteogenesis throughout the healing process. This approach offers a promising direction for improving post-extraction management protocols in dental surgery.

## 1. Introduction

Healing of a tooth extraction socket is a complex, multi-stage process involving hemostasis, inflammation, proliferation, and remodeling. The formation and stabilization of a blood clot within the socket is critical, as it serves as a natural scaffold for cell migration and subsequent tissue regeneration. Disruption of this delicate process can lead to a common and painful postoperative complication known as alveolitis (dry socket). Alveolitis is characterized by inflammation of the alveolar bone, severe pain often radiating to the ear, delayed healing, and potential infection, significantly impairing patient quality of life and necessitating additional clinical interventions [1]. The etiology of alveolitis is multifactorial, involving local, systemic, and iatrogenic components. Key risk factors include premature clot dissolution or displacement, microbial contamination of the socket, traumatic extraction, smoking, pre-existing conditions such as diabetes or immunodeficiencies, and improper postoperative care [2]. Despite standardized preventive measures—including socket debridement, systemic or local antibiotics, and antiseptic rinses [3]. The cornerstone of prevention is maintaining proper oral hygiene and avoiding factors that compromise local immunity, such as smoking, which can delay the healing process. One of the effective methods for preventing complications, including alveolitis, is the use of collagen [4,5]. Collagen is the primary structural protein that forms the foundation of connective tissues, including skin, bones, cartilage, and ligaments [6]. In dentistry, collagen is utilized in various pharmaceutical forms (such as sponges, gels, films, etc.), which can accelerate healing and reduce inflammation [7]. The use of collagen for alveolitis prevention has several advantages and limitations. However, with the appropriate selection of pharmaceutical form, its properties, application conditions, and active components, collagen represents a promising approach in dental practice. Of particular interest is the potential to develop multicomponent, biodegradable collagen-based formulations tailored to the stages of alveolus healing, capable of providing prolonged and targeted therapeutic effects [1]. The aim of this narrative review is to analyze current scientific evidence and substantiate biopharmaceutical requirements for the development of collagen-containing local delivery systems for the prevention of alveolitis after tooth extraction.

## 2. Materials and Methods

This narrative review was conducted to analyze and summarize existing data on the use of collagen-based topical formulations—such as gels and sponges—for the prevention of alveolitis following tooth extraction. The primary objective of this review was to use the current evidence base to establish the rational requirements for the development of advanced, biodegradable, site-specific collagen delivery systems. The primary objective of this review, driven by the limitations of existing single-component topical agents, was to use the current evidence base to establish the rational requirements for the development of advanced, biodegradable, site-specific collagen delivery systems. 

The methodological approach was structured to identify, evaluate, and synthesize relevant scientific literature and clinical data addressing the following aspects: (1) pathogenesis and healing physiology of post-extraction sockets; (2) biological properties of collagen relevant to wound healing; (3) pharmacotechnological characteristics of collagen-based delivery systems; and (4) their clinical effectiveness in alveolitis prevention.

### 2.1. Literature Search Strategy

A comprehensive literature search was conducted using PubMed, Scopus, Web of Science, and Google Scholar databases for the period from 2010 to 2025, as well as fundamental research that forms the basis of the field of knowledge from previous years. 

Given that collagen’s relevance in alveolitis prevention arises from both its local clinical use and its underlying biomaterial properties, the literature search followed a dual-orientation approach:

1. Clinical Focus: studies addressing alveolitis, dry socket prevention, postoperative complications, and clinical outcomes following collagen sponge or gel application.
2. Biopharmaceutical focus: publications discussing dosage forms and properties of collagen matrices that must be considered when developing collagen-containing systems for the prevention of alveolitis.

The following keywords and Boolean combinations were used: “collagen AND alveolitis”, “dry socket prevention”, “collagen sponge OR collagen gel”, “wound healing dental socket”, “collagen drug delivery”, “collagen delivery system”, “collagen AND bone regeneration”, “collagen AND wound healing”, “topical dental formulations”, and “post-extraction inflammation control.” 

### 2.2. Inclusion and Exclusion Criteria

Inclusion criteria were defined to ensure scientific relevance and consistency: original articles published in peer-reviewed journals between 2010 and 2025; studies written in English or with available English translations; clinical trials, in vivo studies, and in vitro experiments investigating collagen-based delivery systems applied locally in the oral cavity; research specifically evaluating the pharmacological, biological, or material properties of collagen in wound healing and alveolitis prevention.

Exclusion criteria: studies focused exclusively on non-collagen biomaterials (e.g., hyaluronic acid, chitosan, synthetic polymers without collagen), research investigating collagen oral administration (*per os*), reviews, meta-analyses, editorials, case reports without original experimental data, and non-English articles with no available full-text translation or institutional access.

All eligible studies were screened based on titles and abstracts, followed by full-text analysis. The selected literature was evaluated for methodological rigor, scientific relevance, and applicability to the review objectives.

### 2.3. Assessment of Evidence Level and Study Quality

Given the narrative nature of this review and the heterogeneity of included studies, a formal quality assessment using standardized tools (e.g., GRADE, Cochrane Risk of Bias) was not systematically applied. However, the methodological characteristics and level of evidence of key studies were considered during data synthesis.

Studies were categorized according to study design:

Randomized controlled trials (RCTs); prospective cohort studies and case-control studies; retrospective studies and case series; in vitro and preclinical studies.

## 3. Healing Physiology

Following tooth extraction, socket healing progresses through four sequential, overlapping stages: hemostasis, inflammation, proliferation, and remodeling [8].

The initial hemostasis phase is critical, as the formation of a stable blood clot seals the wound, protects exposed bone and nerve endings, and provides a provisional extracellular matrix for cell migration. This fibrin-rich scaffold, infused with platelets and growth factors, is essential for initiating subsequent regenerative processes [9].

A controlled inflammatory response follows, typically peaking within 48–72 h, to clear debris and prevent infection [10]. This phase transitions into proliferation, characterized by the influx of fibroblasts and endothelial cells, forming vascularized granulation tissue that replaces the clot over the first 3–4 weeks [9].

The final maturation and remodeling phase involves the gradual replacement of granulation and immature woven bone with mature lamellar bone, a process that continues for months. While significant bone fill occurs within 4–8 weeks, complete alveolar ridge remodeling and stabilization can take 6 months or longer, necessitating long-term evaluation for a full prognostic assessment [8,11,12,13].

## 4. Etiology and Pathogenesis of Alveolitis

**Figure 1:**
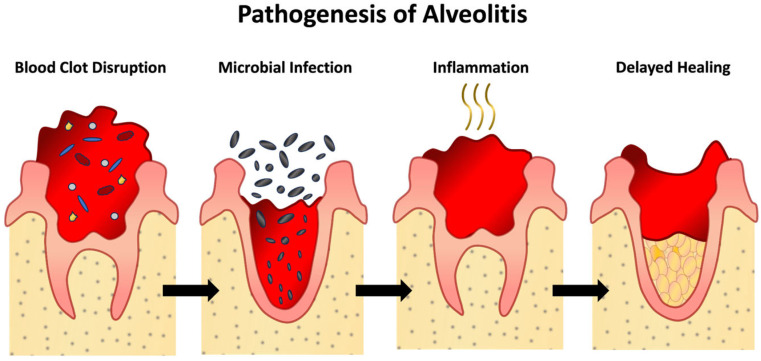
Pathogenesis of Alveolitis.

The exact pathogenesis of alveolitis remains incompletely understood but is thought to involve excessive fibrinolytic activity and impaired microcirculation (Figure 1) [14,15]. The bacterial species implicated in alveolitis development include Prevotellas, Fusobacteria, Peptococci, anaerobes, and Proteus. These microorganisms can colonize the alveolus, trigger inflammation, and delay healing [16].

Human factors also play a critical role in alveolitis etiology. A comparative analysis of two clinics (involving over 2600 patients) revealed higher complication rates following surgical extractions compared to simple extractions [5]. Notably, complication rates varied significantly between institutions, likely due to differences in surgical techniques and operator experience, a key consideration in understanding alveolitis etiology. 

## 5. Collagen as a Pharmaceutical and Dental Material

### 5.1. Sources and Parameters of Collagen Standardization

In scientific and clinical literature, the term “collagen” is used in a broad and sometimes non-specific manner. Most commonly, it denotes type I collagen, the primary structural protein of the extracellular matrix and a key determinant of connective tissue integrity. Collagen is the most abundant protein in the human body and a fundamental component of connective tissues. It is also a major organic constituent of dentin, predominantly represented by collagen types I and III [17,18]. Its characteristic triple-helical structure confers exceptional mechanical strength, elasticity, and biological functionality [17].

To date, approximately 29 types of collagens have been identified. In dentistry and oral-maxillofacial surgery, highly purified collagen forms are most widely used. These include bovine, porcine, marine, allogeneic, and recombinant collagen. Collagen is commonly obtained from natural sources via acid or alkaline enzymatic hydrolysis, followed by multiple stages of product purification [11,17]. The global collagen market in 2025 is estimated to be between USD 4.9 and 6.5 billion, with bovine and porcine collagen continuing to account for approximately 30 percent of this market [19].

Alternative sources are also emerging, such as marine organisms (fish, jellyfish, sponges, sea urchins) [19]. Collagen derived from marine organisms and bovine sources differ in their amino acid composition, which results in distinct physico-mechanical properties. This is a critical consideration when developing materials and products for dentistry based on these collagens. It is important to note that even collagen obtained from the same type of animal raw material can vary significantly in amino acid content depending on its habitat. This variation influences the mechanical strength and elasticity of the resulting materials [20]. Research indicates that specific amino acids can distinctly influence the mechanical properties of collagen; for instance, serine is associated with elasticity, while proline and hydroxyproline affect the denaturation temperature, among other characteristics [21].

Therefore, the standardization of animal-derived and marine collagen remains a key challenge due to variations in source and extraction methods, which can affect the consistency and quality of the final product [11,17].

The most recent contemporary alternative to classical animal collagen is recombinant human collagen (RHC), produced using biotechnological methods. Its production process allows for the programming of the desired amino acid composition and mechanical properties of the resulting products. Furthermore, it mitigates the risks of zoonotic infection transfer inherent in collagen derived from animal raw materials and avoids the negative organoleptic properties (a frequent problem with marine collagen), thereby creating a highly standardized substance [22].

A global challenge for genetically engineered collagen lies in the cost of its production process. However, recent publications highlight novel approaches capable of significantly improving the cost-effectiveness of manufacturing [23]. All described collagen types demonstrate biocompatibility with human tissues [11,17].

The standardization of collagens, regardless of their source or production method, is based on key critical points, which are grouped into physico-chemical, biological, and technological properties (Table 1). From a biological safety perspective, collagen as an excipient for medicine, and dentistry in particular, must lack immunogenicity, cytotoxicity, and pyrogenicity.

As a scaffold and delivery system, collagen is used in various configurations—including sponges, gels, membranes, and composite systems—each of which influences both the application method and the functional (technological) properties of the material [11,25,26,27]. This will be discussed further below.

Composite collagen-based systems integrate collagen with additional materials to enhance structural and therapeutic performance [17,25]. These include porous hydroxyapatite–collagen composites that mimic the architecture of natural bone [28], collagen sponges combined with bone substitutes such as allografts or xenografts for alveolar ridge preservation [26], as well as collagen scaffolds loaded with bioactive agents—including antibiotics and growth factors—to provide antimicrobial activity or stimulate regenerative processes. The selection of an appropriate collagen-based system for dental applications requires careful consideration of several critical factors: the biomaterial’s interfacial interactions with the target site and its structural evolution following administration and throughout the healing period, as well as its compatibility with potential incorporated bioactive agents.

The structural characteristics of collagen are determined by processing techniques, resulting in either preservation of the native triple-helical conformation, denatured states, or cross-linked configurations exhibiting enhanced resistance to enzymatic degradation. Furthermore, advanced collagen matrices may be engineered to incorporate various therapeutic compounds or biologically active constituents. Gel forms do not form a volumetric framework, have low rigidity, but have the ability to swell and the possibility of administration by injection, which is manifested in the correction of the form after administration [29]. Thus, the technology to produce a particular dosage form not only determines its biopharmaceutical properties, but also the range of active ingredients used depends on compatibility, their mode of release and clinical applicability. Moreover, the choice of the form should take into account the characteristics of the healing stage, the target area of application, and oral conditions, including moist environment, pressure, tissue mobility and microbial load, pH, and other factors. In recent years, significant research has focused on investigating and controlling the gelation process in collagen solutions. It is thought that this could potentially allow for the creation of composites with tailored physical, chemical, and biological properties, thereby broadening their biomedical applications through innovative delivery methods and enhanced therapeutic efficacy [30].

### 5.2. Mechanism of Action in Alveolar Healing

**Figure 2:**
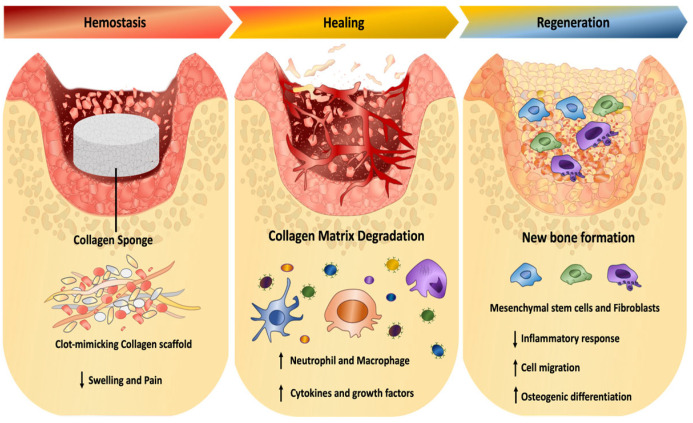
Collagen in the alveolar healing process.

Collagen plays an active role in alveolar healing by providing structural support and forming a provisional extracellular matrix that facilitates cellular adhesion, migration, and proliferation. Specific amino acid sequences within collagen fibers, such as Arg-Gly-Asp (RGD), are recognized by integrins on fibroblasts, osteoblasts, and endothelial cells, activating signaling pathways involved in angiogenesis and extracellular matrix restoration—key processes in post-extraction tissue regeneration [31]. The mechanism of action of collagen material in the alveolar healing process is shown in Figure 2.

In addition, collagen exhibits moderate antimicrobial and anti-inflammatory activity, contributing to reduced infection risk and postoperative discomfort. In dental applications, collagen membranes and gels stabilize the blood clot and protect it from mechanical and microbial disruption, preventing premature clot loss [12].

### 5.3. Formulations and Products for Medical Applications Based on Collagen and Their Key Biopharmaceutical Characteristics

Collagen can be formulated into various dosage forms, including sponges, gels, films, and solutions (including stimulus-sensitive systems), while nanosystems, microspheres, and injectable gels are used less frequently. Fabrication technology largely determines the physicochemical and morphological properties of these systems and should be tailored to the wound type, as requirements for alveolar applications differ from those for other wound indications (e.g., diabetic wounds) [11,27]. Lyophilization is commonly employed to produce porous collagen sponges with a stable three-dimensional structure, high absorbency, mechanical integrity, and the capacity to retain biologically active substances [24]. In alveolar sockets, resorbable collagen sponges act as temporary scaffolds for cell migration, support hemostasis, and form a barrier against infectious agents [25]. Considering the phases of alveolar wound healing, a functional biodegradation period of approximately 7–14 days appears optimal for non–cross-linked collagen sponges. During the first 1–3 days after tooth extraction, hemostasis and blood clot formation dominate, with collagen providing mechanical protection of the wound surface [11]. From days 3 to 7, fibroblast and endothelial cell migration, granulation tissue formation, and angiogenesis occur, during which collagen serves as a provisional extracellular matrix. By days 10–14, epithelialization and early tissue remodeling begin, reducing the need for scaffold persistence. Prolonged presence of the biomaterial may impede cellular infiltration, disrupt tissue architecture, and potentially favor microbial colonization. Thus, biodegradation within 7–14 days allows collagen to exert protective and regenerative functions without interfering with physiological healing.

Reported residence times vary depending on material composition and cross-linking. Absorbable type I/III collagen sponges (e.g., Ateloplug) are reported to resorb within approximately 2–4 weeks [25], while lyophilized collagen sponges used in endodontics (e.g., Hemospon^®^ (Maquira, Brazil) have been associated with trabecular bone formation and pronounced vascularization at around 24 days [32,33]. In preclinical rat models, collagen implants are typically detectable at day 7 but largely resorbed by day 28, with osteoid formation observed from two weeks and progressive mineralization over several months, supported by the collagen scaffold [32,33]. In contrast, cross-linked collagen materials are designed for extended persistence; for example, ribose-cross-linked sponges used for ridge preservation maintain structural integrity for approximately 16–24 weeks, with sparse histological remnants at six months [26,32].

Overall, these data do not define a single universal biological optimum for collagen residence time. For non–cross-linked collagen scaffolds intended primarily to prevent alveolitis, a functional presence ranging from approximately 7 days to no more than one month appears acceptable, with around 14 days representing a reasonable compromise. Other clinical indications, such as ridge preservation using cross-linked matrices, require distinct biodegradation profiles that should be considered as separate product design objectives.

Biopharmaceutical characteristics of possible collagen-based formulations depending on the dosage form are presented in Table 2. The values presented are based on data from analyzed scientific studies and publications. However, it should be noted that the values of the listed characteristics are not officially approved and are determined based on clinical necessity.

### 5.4. Clinical Applications 

Clinical evidence supports the efficacy of collagen-based materials in reducing postoperative complications following tooth extraction. A retrospective analysis of more than 2600 third molar extractions using type I collagen sponges reported an overall complication rate of 2%, including 3.00% infectious complications and only 1.14% alveolitis, indicating a favorable safety and efficacy profile [5]. These findings are consistent with the ability of collagen sponges to stabilize the extraction socket during early healing and to support physiological regeneration processes, thereby reducing the risk of inflammation and alveolitis.

The preventive potential of collagen sponges was further confirmed in a randomized controlled “split-jaw” trial published in 2020 [25]. In this study, 36 patients with symmetrical retained lower third molars received an absorbable collagen sponge (Ateloplug^®^) in one socket, while the contralateral site served as a control. The collagen-treated sites showed significantly lower pain scores during the first postoperative week and reduced periodontal probing depth by the second week, reflecting improved early soft tissue healing. Although radiological bone regeneration at 14 weeks did not differ significantly between groups, enhanced early recovery and pain reduction may contribute indirectly to lowering the risk of alveolitis.

At the same time, collagen sponges lacking antimicrobial components do not inherently suppress interaction with oral biofilm. Several studies have demonstrated the effectiveness of systemic or local antibiotic therapy in preventing postoperative infections and alveolitis [41], suggesting that combined or multilayer dosage forms incorporating antimicrobial agents may further enhance clinical outcomes.

In parallel, current research increasingly focuses on the biofunctionalization of collagen films to enhance their regenerative capacity. While collagen films are traditionally used as barrier membranes for guided bone and soft tissue regeneration, their effectiveness can be significantly increased by loading them with biologically active substances. In particular, the use of bone conditioned medium (BCM) derived from autogenous bone has been shown to stimulate osteoblast proliferation, differentiation, and mineralization, accelerating bone regeneration and reducing inflammatory complications, including alveolitis [42]. These approaches highlight the potential of bioactivated collagen-based systems to improve the microenvironment of the extraction socket and to support physiological healing mechanisms.

Collagen hydrogels have been used in medical practice since the mid-20th century. In recent research, they are increasingly being applied in dentistry for dentin regeneration [18,43]. Within composite matrices, collagen serves as a component in the development of modern stimuli-responsive systems—for example, thermosensitive hydrogels and growth factor carriers. According to the researchers’ design, these are introduced into the extraction socket in liquid form and subsequently undergo gelation to form a hydrogel [44]. Table 3 presents the characteristics of several well-known and clinically effective collagen-based products. As evident from the table, the majority of manufactured collagen products are in sponge form.

### 5.5. Limitations and Safety Considerations 

Despite its favorable biological properties, collagen-based therapy has several limitations that must be considered in clinical practice [11]. Effective application requires proper socket debridement and control of pre-existing inflammation. In cases of deep infection, collagen preparations alone may be insufficient without appropriate antibiotic therapy [45,46]. In addition, hypersensitivity or allergic reactions to collagen, although relatively rare, remain an important limitation and are consistently listed as exclusion criteria in clinical and preclinical studies [47,48].

The immunogenicity of collagen depends on its source and processing. Mammalian-derived collagen generally carries higher immunogenic and pathogen-related risks compared with marine or thoroughly deantigenized materials [49]. To mitigate these risks, manufacturers apply additional purification steps, such as enzymatic removal of telopeptides in porcine atelocollagen (Ateloplug^®^) or the use of deantigenized equine collagen (Salvecoll E gel) [25,50].

The oral environment itself presents significant challenges for topical therapies aimed at alveolitis prevention. Abundant salivation, enzymatic activity, and the presence of salts can rapidly degrade or inactivate active components of collagen-based preparations, particularly during the critical early postoperative period [51]. Mechanical factors, including eating, may further dislodge the material from the socket, reducing the duration of its therapeutic action [52]. These factors underscore the importance of controlled oral hygiene and minimization of mechanical stress to ensure adequate early exposure of the wound to the therapeutic agent.

Another major limitation is the formation of oral biofilm. Biofilm acts as a protective microbial barrier that impedes the penetration of active substances into tissues and shields microorganisms from antimicrobial agents [53]. Contact between collagen materials and biofilm may reduce therapeutic efficacy, promote degradation of the collagen matrix, and impair normal healing [54].

Finally, many topical agents targeting the early stage of blood clot formation may have limited effectiveness due to insufficient residence time. Rapid, single-phase degradation may prevent sustained therapeutic exposure during the critical phases of healing [55]. Given the central role of the blood clot in alveolar repair, its premature disruption can lead to infection and inflammation, emphasizing the need for dosage forms with controlled residence time and degradation profiles [11]. A concise comparison of the principal advantages and limitations associated with collagen application for alveolitis prevention is presented in Table 4. 

## 6. Discussion

Materials used for alveolitis prevention differ in mechanism of action, clinical efficacy, safety, cost, and patient-related outcomes. Collagen-based systems are widely applied as scaffolds and clot stabilizers, occupying an intermediate position between pharmacological approaches (antibiotics, antiseptics) and alternative biomaterials or physical interventions [17,27,34,55].

### 6.1. Evidence Quality and Methodological Considerations

The body of evidence supporting collagen-based interventions for alveolitis prevention is heterogeneous in terms of study design, outcome measures, and methodological rigor.

High-quality evidence is available from:

A split-mouth randomized controlled trial (n = 36) evaluating absorbable collagen sponges (Ateloplug^®^), which demonstrated significant reduction in pain and improved soft tissue healing during the first two postoperative weeks [25]. This study employed appropriate randomization, blinding of outcome assessors, and validated pain scales, representing Level 1b evidence according to the Oxford Centre for Evidence-Based Medicine.

Moderate-quality evidence includes:

A large retrospective cohort study (n > 2600) reporting low complication rates (1.14% alveolitis) following collagen sponge use [5]. While the sample size is robust, the retrospective design and lack of a control group limit causal inference (Level 3 evidence).

Comparative studies on PRF/PRP show reduced alveolitis incidence (6% vs. 20%) [55], though these often lack standardized protocols and long-term follow-up.

Limitations identified across studies:

Many clinical trials are underpowered due to small sample sizes (n < 50);

Lack of standardized definitions for alveolitis and healing outcomes;

Short follow-up periods (typically ≤14 days), insufficient to assess bone regeneration;

Variability in collagen source, processing, and formulation, limiting comparability;

Absence of patient-reported outcome measures in some studies;

Potential publication bias favoring positive results;

Preclinical and in vitro studies provide valuable mechanistic insights into collagen’s role in hemostasis, cell adhesion, and scaffold function, but translation to clinical outcomes requires validation in well-designed human trials.

Overall assessment:

While existing evidence supports the safety and potential efficacy of collagen-based systems in reducing postoperative complications, the quality of evidence remains moderate to low for most outcomes. High-quality, adequately powered, multicenter RCTs with standardized protocols and long-term follow-up are needed to establish definitive clinical recommendations.

### 6.2. Biopharmaceutical Properties

Collagen combines biodegradability, biocompatibility, and technological versatility. Its degradation profile can be modulated by controlled cross-linking; ribose-cross-linked collagen sponges maintain structural integrity for approximately 16–24 weeks while remaining permeable to progenitor cell migration and resistant to early degradation in the oral cavity [26,56]. Established processing strategies reduce immunogenicity and enhance biocompatibility [49,57].

Available as sponges, gels, and membranes, collagen enables multilayer architectures with tailored porosity and permeability. Pore sizes of approximately 100–500 µm support osteoblast migration, clot stabilization, and extracellular matrix–like function [25,35]. Collagen sponges are widely used for socket sealing, stabilizing the graft and blood clot while limiting soft-tissue ingrowth [58,59]. Multilayer designs combining porous and denser components have therefore been proposed to optimize biological integration and mechanical protection [60,61].

Collagen matrices also support controlled drug delivery. Hydroxyapatite–collagen composites (≈75 m^2^/g) can adsorb antimicrobials such as vancomycin and maintain antibacterial activity for up to 14 days [62], while collagen–gelatin scaffolds enable sustained release of growth factors (e.g., bFGF) during early healing [63]. This multifunctionality may reduce formulation complexity and improve risk management. In some indications, ribose-cross-linked collagen sponges eliminate the need for additional barrier membranes [26,56], and long-lasting injectable systems (e.g., PMMA–collagen gels) provide predictable structural support [47].

### 6.3. Cost, Availability, and Patient-Centered Considerations

Collagen products are generally associated with higher costs due to raw material quality requirements, purification, quality control, and processes such as lyophilization [11,26,27], although lower-cost derivatives such as gelatin are available [35]. Recombinant human collagen remains limited by production costs and yield [7,17]. In contrast, antibiotics and antiseptics are widely available and relatively inexpensive [2], while PRF/PRP require on-site equipment and trained personnel [64]. Physical methods such as Kinesio Tape are low-cost and non-invasive [65].

From a patient perspective, collagen-based materials are associated with improved postoperative comfort. Absorbable collagen sponges significantly reduce pain during the first postoperative week [5,22], comparable to the benefits reported for PRF/PRP in reducing pain and swelling [14,25,64], while Kinesio Tape improves quality of life by reducing trismus and pain [10,65]. Conversely, systemic antibiotics may cause adverse effects [65], and some older topical socket dressings have been linked to foreign body reactions and nerve dysesthesia [46].

### 6.4. Complications and Risk Profile

Collagen-based systems reduce inflammatory complications by stabilizing the blood clot and supporting early healing [5]. Retrospective data report a low alveolitis incidence (approximately 1.14%) following collagen plug use [5,66]. However, risks include hypersensitivity reactions [11], potential zoonotic transmission from mammalian sources [45], and limited efficacy in contaminated wounds without adjunctive therapy [11].

Autologous PRF/PRP demonstrates a marked reduction in alveolitis incidence (e.g., 6% versus 20% in controls) with minimal immunological risk [64]. Antibiotics effectively reduce postoperative infections and dry socket, particularly amoxicillin–clavulanic acid, but raise concerns regarding antimicrobial resistance [2,10,14,41,46]. Topical chlorhexidine significantly reduces alveolitis incidence [10,14] but carries a documented risk of anaphylaxis, prompting recommendations to favor saline irrigation in certain protocols [14].

### 6.5. Potential for Developing Specific Formulations

Given the current known range of collagen-based preparations and medical products for use in dentistry, the development of a new drug delivery system requires a scientifically grounded approach to selecting the dosage form.

**Collagen sponges** completely fill the alveolar socket, providing reliable protection for the forming blood clot against mechanical impact from food and saliva, which helps to reduce the risk of infection [4,5,25]. They are capable of absorbing a large volume of fluid and promote the formation of granulation tissue [5,7,11,66]. They can also serve as a delivery system for antiseptic and antibacterial agents; the release of active substances from the sponges is prolonged, likely occurring through erosion and diffusion [7,55,66]. They are capable of biodegrading over time and do not require removal. As carriers for medicinal substances, they hold a significant advantage over gels—namely, being a solid, pre-formed dosage form [5,25].

**Collagen hydrogels** represent soft and non-traumatic dosage forms [7,11]. Due to their high moisture content, hydrogels promote healing [7,11,45]. Their dosing by volume facilitates their successful use as carriers for active compounds from various pharmacological classes [7,11,29,66]. In contrast to collagen sponges, collagen-based hydrogels typically provide immediate release of active components, with only a very limited capacity for prolonging the effect [7,11]. Through the gradual dissolution of the gel in the socket, the release of the active component is thought to be more complete [7,55,66].

**Collagen membranes**, unlike collagen-based sponges and hydrogels, seldom function as standalone carriers for active components [7,17,42]. Their primary function is to act as a barrier, protecting the tooth socket and any material placed within it from external factors [34,42]. The placement of a collagen membrane aids in forming and maintaining a stable volume of blood clot and granulation tissue [67]. The most frequently applied approach currently involves combining several collagen-based preparations or medical products [55,66]. For instance, collagen sponges may be used in combination with collagen hydrogels, and the placement of a sponge in the socket may be accompanied by coverage with a membrane [11,25,27].

Developing innovative collagen-based drugs for dentistry, particularly for alveolitis, would likely require combining the advantages of these forms. Today, a similar effect may be achievable through the use of stimuli-responsive delivery systems, for example, those administered as a gel that forms a solid depot directly on the mucosa (in situ) [7,29,68].

### 6.6. QbD Framework for the Development of Collagen-Based Local Delivery Systems 

The Quality by Design principles defined in ICH Q8 provide a structured basis for designing collagen-based materials with predictable performance in the post-extraction socket [69,70,71,72]. Applying this approach helps align formulation properties with the biological phases of healing and the specific constraints of the oral environment [73]. The limitations identified in existing collagen preparations, such as rapid degradation and insufficient functional stability, indicate the need for more controlled and robust material design. The following sections summarize the target product profile and the critical quality attributes required for such a system. 

#### 6.6.1. Quality Target Product Profile (QTPP) for a Collagen-Based Socket Healing System 

Based on the clinical and material science evidence summarized in this review, the QTPP for a collagen-based system used in the prevention of alveolitis includes Table 5.

The QTPP therefore provides a clear, clinically grounded target for the development of a collagen-based medicine that is biologically active, technologically consistent, and aligned with ICH Q8 expectations [39,58,59,60,63]. 

#### 6.6.2. Critical Quality Attributes (CQAs) of Collagen Matrices for Alveolitis Prevention 

To achieve the QTPP defined above, several Critical Quality Attributes must be controlled during development and manufacturing. These attributes directly influence the safety and clinical performance of the collagen matrix [70,71,73] as outlined in Table 6.

These CQAs ensure that the final product consistently achieves the therapeutic functions required for predictable socket healing and effective prevention of alveolitis, in accordance with the foundational concepts of ICH Q8 [73].

## 7. Conclusions

### 7.1. Limitations of Current Topical Approaches

Topical agents used in the prevention and therapy of alveolitis face several limitations that reduce their long-term effectiveness. Current strategies typically combine local preparations with adjunctive measures such as systemic or local antibiotic therapy and strict oral hygiene. However, many topical formulations exhibit rapid degradation and insufficient activity throughout the sequential stages of post-extraction healing, particularly with respect to bone tissue regeneration [29,66].

### 7.2. Collagen-Based Preparations in Post-Extraction Management

Collagen-based dosage forms—including sponges, gels, and membranes—demonstrate high potential for inclusion in postoperative management protocols following tooth extraction, especially in patients at increased risk of complications such as alveolitis. Their effectiveness, however, is strongly dependent on appropriate selection of dosage form, adherence to sterility requirements, adequate socket debridement, and, when indicated, combination with antimicrobial or anti-inflammatory therapy.

Current single-layer collagen preparations are often unable to provide sustained and stage-specific therapeutic activity, limiting their ability to fully support the complex biological processes involved in alveolar healing.

### 7.3. Stage-Dependent Therapeutic Requirements

Analysis of the post-extraction healing process indicates that effective prevention of alveolitis requires a dosage form capable of addressing distinct biological challenges at different stages of healing.

#### 7.3.1. Early Stage (Hemostasis and Clot Stabilization)

The primary requirement is stabilization of the blood clot and protection against premature degradation, which is critical for successful alveolar healing.

#### 7.3.2. Intermediate Stage (Infection Risk)

As microbial contamination becomes a major concern, the preparation should exhibit antibacterial activity to reduce the risk of alveolitis and other inflammatory complications.

#### 7.3.3. Late Stage (Tissue Regeneration)

During active regeneration, therapeutic focus should shift toward stimulation of collagenogenesis and osteogenesis to ensure proper bone restoration and alveolar structuring.

Existing formulations rarely provide adequate functionality across all these stages, highlighting the need for more advanced delivery systems.

### 7.4. Rationale for Multilayer Biodegradable Systems

The development of a biodegradable multilayer collagen-based preparation represents a promising strategy to overcome current limitations. Such systems can be designed to deliver stage-appropriate therapeutic effects while gradually degrading in parallel with tissue healing. This approach enables prolonged and controlled release of active agents, prevents accumulation of residual material, and minimizes the risk of adverse effects.

By integrating multiple functional layers, a single preparation may simultaneously **provide** early hemostatic support, intermediate antimicrobial protection, and late-stage regenerative stimulation, thereby enhancing overall clinical effectiveness and accelerating tissue recovery after tooth extraction.

### 7.5. Implications for Formulation Design and Quality by Design (QbD)

The analysis presented in this review demonstrates that while collagen-based systems are highly promising for alveolitis prevention, currently available rapidly degradable and single-layer products do not adequately address the sequential biological requirements of the post-extraction socket. Applying the principles of ICH Q8 and a structured Quality by Design (QbD) framework, this study proposes a preliminary Quality Target Product Profile and defines Critical Quality Attributes specific to collagen matrices intended for this indication.

Key CQAs—such as controlled degradation kinetics, mechanical stability in the moist oral environment, and compatibility with antimicrobial or regenerative agents—extend conventional requirements for semi-solid and sponge-like dosage forms and support a more precise definition of performance expectations.

### 7.6. Future Development Directions

Future research and development should focus on several priority areas:

Engineering multilayer or multiphasic collagen systems capable of adapting their function to inflammatory, proliferative, and early osteogenic phases.

Optimization of crosslinking strategies and composite architectures to balance early structural support with timely resorption and minimal interference with tissue remodeling.

Standardization of biopharmaceutical performance, including pore architecture, fluid absorption, and stability under salivary enzymatic conditions.

Development of in vitro–in vivo correlation models specific to oral wound environments to improve prediction of degradation behavior, antimicrobial efficacy, and cell–matrix interactions.

### 7.7. Overall Perspective

Collectively, these findings outline a clear roadmap for the development of next-generation collagen-based delivery systems capable of staged therapeutic action, improved predictability, and enhanced clinical value in the prevention of alveolitis and other post-extraction complications.

## Figures and Tables

**Table 1:** Selected physico-chemical properties for collagen standardization.

Standardization Parameter	Critical Quality Value	Analytical Methods	Literature
Physico-chemical Properties			
Amino acid composition	Conformity to the profile of a specific collagen type; absence of impurities	HPLC with various detection modes	[11]
Hydroxyproline content	Quantitative determination of collagen content	Calorimetry	[11,24]
Stability of the three-dimensional structure	Preservation of the native structure, crucial for biological (therapeutic) function	Various spectroscopy techniques	[11]
Denaturation temperature	Indicator of stability	Calorimetry	[6,11]
pH	Critical for stability and biocompatibility of the preparation	Potentiometry	[24]
Viscosity	An important parameter for subsequent manufacturing of collagen-based materials	Various rheometry techniques	[24]

**Table 2:** Biopharmaceutical characteristics of collagen-based products depending on their dosage form.

Parameter	Optimal Value	Literature
Collagen Sponges		
Biodegradation	Depending on the application and type of collagen—from 7 days to 6 months	[26,32,34]
Porosity	≥80%, pore size 50–200 µm	[35]
Water Absorption	High (for exudate absorption)	[35]
Swelling Capacity	Limited	
Cell Adhesion	Moderate	[24]
Collagen Membranes		
Cell Adhesion	High (for better proliferation)	[36,37,38]
Hydrophilicity	The angle of contact with water is less than 45°, which affects the interaction between the membrane material and liquids and host cells during implantation.	
Strength/Elasticity	5–15 MPa/50–100%	
Biodegradation	8–12 weeks	
Collagen Gels		
Rheological properties	Pronounced viscoelastic behavior (storage modulus G′ and loss modulus G″), with mechanical characteristics comparable to those of dental pulp tissue, indicating high biorelevance.	[39]
pH	A pH range of 6.5–7.4, which is sufficient for the formation of a stable collagen gel after neutralization and falls within the physiological range, ensuring biocompatibility and the absence of irritant or cytotoxic effects.	[40]

**Table 3:** Selected medical collagen-based products for application in dentistry.

Product (Trade Name)	Composition	Dosage Form	Collagen Type	Country of Origin
Alvanes^®^	Lyophilized collagen, lidocaine, iodoform, chlorhexidine and metronidazole / lincomycin	Sponge	Type I	Russia
Alvocon	Solution of animal-derived collagen, calcium hydroxyapatite, iodoform, metronidazole	Sponge	From cattle hide splits	Russia
Alvostaz	Tricalcium phosphate, olive oil, eugenol, iodoform, collagen	Sponge	From cattle hide splits	Russia
Atelocollagen Cone	Collagen	Sponge	Type I	Republic of Korea
Collacone^®^/Bio-Oss Collagen	Natural porcine-derived collagen	Sponge	From pork collagen.	Germany
Collost^®^	Collagen, Glucose solution	Gel	Type I	Russia
Coloplug	Bovine collagen	Sponge	Type I	India
Creos™ Xenoplug	90% Xenogeneic bone mineral, 10% Porcine collagen	Sponge	Type I	Switzerland
DSI Sponge Plus	Collagen (base), iodoform, eugenol, thymol, and lidocaine	Sponge	Type I	Israel
HealiAid^®^	Collagen	Sponge	Type I	Taiwan/China
Helistat^®^	Type I collagen from bovine tendon (100% bovine collagen)	Sponge	Type I	USA
Hemocollagène^®^	Non-denatured, lyophilized collagen	Sponge	Type I	France
Micro-tappers	Collagen, sanguiritrin, lidocaine	Sponge	Self-absorbing cones made of highly purified bovine Type I collagen	Russia
Mucograft Seal^®^	Native collagen derived from porcine raw material	Sponge	Types I and III	Switzerland
PARASORB^®^ Cone/Parasorb^®^ Cone Genta	Native equine collagen fibers, gentamicin sulfate	Sponge (cone)	From horse tendons	Germany
Teruplug^®^	Atelocollagen	Sponge	Type I	Japan

**Table 4:** Advantages and Disadvantages of Collagen Use in the Prevention of Alveolitis.

Advantages	Disadvantages
Promotes the regeneration of damaged tissues	Requires proper decontamination of the socket prior to application
Accelerates the healing process	May be ineffective in cases of deep or extensive infections
Supports the preservation of alveolar socket architecture	Risk of zoonotic disease transmission and the need for routine screening of livestock for infectious agents
Decreases the risk of inflammatory complications	High costs associated with cleaning procedures and technical challenges in formulation and application

**Table 5:** Characteristics of collagen matrix for alveolitis prevention and socket healing.

Parameter	Specification
Intended use	Local prevention of alveolitis and support of physiological post-extraction socket healing
Target population	Adults undergoing simple or surgical tooth extraction, including patients at increased risk of postoperative complications
Route of administration	Local placement directly into the extraction socket
Dosage form	Biodegradable collagen matrix (e.g., sponge, sponge with protective membrane, or structured multi-component scaffold) intended for single use
Mechanism of action	Stabilization and protection of the blood clot; formation of a temporary extracellular matrix supporting cellular migration; reduction in local irritation; facilitation of angiogenesis and early bone tissue formation; optional incorporation of antimicrobial or regenerative agents
Degradation profile	Predictable biodegradation within 7–14 days, corresponding to inflammatory and early proliferative phases of socket healing
Biopharmaceutical properties	High porosity with pore sizes of approximately 50–200 μm; effective absorption of wound exudate; structural stability in the moist oral environment
Safety profile	High biocompatibility; low immunogenic potential; sterility; absence of transmissible agents
Performance outcomes	Reduction in postoperative pain; decreased incidence of alveolitis; improved soft tissue healing and early bone regeneration
Usability	Simple intraoperative placement; no need for removal; compatibility with standard dental surgical workflow

**Table 6:** Characteristics and justification of requirements for collagen matrices in soft tissue regeneration.

Attribute	Specification and Rationale
Source and purity of collagen	Use of highly purified type I or type I/III collagen with minimal residual immunogenic components; purity directly affects biocompatibility, safety, and the risk of hypersensitivity
Porosity and pore size distribution	Overall porosity above 80% with uniform pore sizes in the range of approximately 50–200 μm to support cellular infiltration, nutrient diffusion, and granulation tissue formation
Level of structural modification	Controlled degree of structural stabilization to ensure biodegradation within 7–14 days; excessive stabilization may delay tissue remodeling, whereas insufficient stabilization may lead to premature disintegration
Mechanical stability in the oral environment	Maintenance of matrix integrity during the first postoperative days despite exposure to saliva and mechanical stress
Absorption and swelling properties	Capacity to absorb wound exudate without structural collapse, maintaining a stable and moist healing interface
Sterility and microbial safety	Validated sterilization process and low initial bioburden to minimize the risk of postoperative infection
Compatibility with active substances	Chemical and physical stability of the matrix upon incorporation of antimicrobial or regenerative agents, ensuring uniform distribution within the material
Release characteristics (medicated matrices)	Initial rapid release during early postoperative hours followed by sustained delivery over several days, when clinically justified
Storage stability	Preservation of pore structure, mechanical integrity, and functional properties throughout the defined shelf life

## Data Availability

No new data were created or analyzed in this study.
